# Benzo[a]pyrene exacerbates allergen-induced airway inflammation through NLRP3-dependent dendritic cell activation and pathogenic T helper cell polarization

**DOI:** 10.3389/fimmu.2025.1699886

**Published:** 2025-11-19

**Authors:** Huamei Zou, Jiaqi Duan, Yongmin Xie, Ruien Chen, Yanyu Ye, Aizhi Zhang, Pingchang Yang, Gui Yang, Xiaoyu Liu

**Affiliations:** 1 Department of Otolaryngology of Longgang Central Hospital & Clinical Collage of Guangzhou University of Chinese Medicine, Shenzhen, China; 2 Institute of Allergy & Immunology of Shenzhen University, State Key Laboratory of Respiratory Diseases Allergy Division at Shenzhen University, and Shenzhen Key Laboratory of Allergy & Immunology, Shenzhen, China; 3 Department of Critical Care Medicine, Second Hospital of Shanxi Medical University, Taiyuan, China

**Keywords:** benzo[a]pyrene, house dust mite, asthma, airway inflammation, dendritic cells, NLRP3 inflammasome, Th2, Th17

## Abstract

**Background:**

Environmental pollutants are known to aggravate allergic diseases, but the molecular mechanisms by which polycyclic aromatic hydrocarbons such as benzo[a]pyrene (BaP) potentiate allergic airway inflammation remain poorly understood.

**Objective:**

We investigated how BaP co-exposure modifies house dust mite (HDM)–driven allergic airway responses, focusing on the role of the NLRP3 inflammasome in dendritic cells (DCs).

**Methods:**

Mice were sensitized and challenged intranasally with HDM with or without BaP. Airway hyperresponsiveness (AHR), bronchoalveolar lavage (BAL) cell counts, lung histopathology, and serum HDM-specific IgE were assessed. Cytokine production and epithelial alarmins were measured by ELISA. The role of NLRP3 was evaluated using Nlrp3^−^/^−^ mice, *in vitro* bone marrow–derived DC (BMDC) cultures, and adoptive transfer of lung DCs. T helper cell polarization was analyzed in OT-II co-culture assays.

**Results:**

Co-exposure to BaP and HDM markedly exacerbated airway inflammation, with enhanced AHR, increased eosinophil and neutrophil infiltration, severe goblet cell hyperplasia, and elevated HDM-specific IgE. Cytokine analysis revealed synergistic induction of Th2 (IL-4, IL-5, IL-13) and Th17 (IL-17A) responses, alongside increased epithelial alarmins (TSLP, IL-33). This exacerbated phenotype was abolished in Nlrp3^−^/^−^ mice, which failed to produce IL-1β/IL-18 and exhibited attenuated inflammation. *In vitro*, BaP synergized with HDM to activate NLRP3 in BMDCs, leading to caspase-1 cleavage, IL-1β release, and enhanced CD80/CD86 expression. Adoptive transfer of BaP/HDM-exposed WT lung DCs, but not Nlrp3^−^/^−^ DCs, was sufficient to drive allergic airway inflammation in naïve recipients. Finally, BaP–conditioned WT DCs skewed naïve CD4^+^ T cells toward Th2 and Th17 lineages, an effect absent in Nlrp3^−^/^−^ DCs.

**Conclusion:**

BaP amplifies allergic airway disease by activating the NLRP3 inflammasome in DCs, thereby enhancing DC maturation, cytokine release, and pathogenic Th2/Th17 polarization. These findings identify a critical mechanism linking environmental pollutants to exacerbated allergic asthma and highlight the NLRP3 inflammasome as a potential therapeutic target.

## Introduction

Asthma is a chronic inflammatory airway disease characterized by airway hyperresponsiveness, mucus overproduction, and infiltration of eosinophils and other immune cells (1). While genetic predisposition contributes to susceptibility, environmental exposures are major determinants of asthma onset and severity. Epidemiological studies have consistently linked urban air pollution with increased asthma prevalence, exacerbations, and hospitalizations (2, 3). Polycyclic aromatic hydrocarbons (PAHs), including benzo[a]pyrene (BaP), are key components of traffic-related air pollution and cigarette smoke, yet their precise immunological mechanisms in asthma pathogenesis remain incompletely defined (4, 5).

Allergic asthma is classically associated with type 2 (Th2) immune responses, characterized by interleukin (IL)-4, IL-5, and IL-13 production, IgE class-switching, and eosinophilic airway inflammation (6, 7). However, a subset of patients with severe or steroid-resistant asthma exhibits mixed granulocytic inflammation, with contributions from Th17 cells and neutrophilic infiltration (8, 9). Environmental pollutants have been proposed as drivers of this endotype, but the cellular and molecular pathways that link pollutant exposure to Th2/Th17 responses remain unclear.

The airway epithelium serves as the first line of defense against inhaled allergens and pollutants. Upon injury or stress, epithelial cells release alarmins such as thymic stromal lymphopoietin (TSLP) and IL-33, which activate dendritic cells (DCs) and promote type 2 immunity (10, 11). DCs are central antigen-presenting cells that integrate environmental signals to shape T cell polarization (12). Whether DCs directly sense PAHs such as BaP and how this influences their immunostimulatory capacity in the context of allergen exposure is not known.

Recent work has identified the NLRP3 inflammasome, a cytosolic multiprotein complex that drives caspase-1 activation and maturation of IL-1β and IL-18, as an important regulator of allergic airway inflammation (13). Activation of NLRP3 has been observed in both innate immune cells and airway structural cells during asthma, but its role in linking environmental pollutants with adaptive immune responses has not been addressed. Given that PAHs can generate oxidative stress and cellular damage, they may act as potent triggers of inflammasome activation.

Here, we investigated the immunological consequences of co-exposure to BaP and house dust mite (HDM), a clinically relevant aeroallergen. We hypothesized that BaP amplifies HDM-induced allergic airway disease by activating the NLRP3 inflammasome in DCs, thereby enhancing Th2/Th17 polarization and driving severe airway inflammation. Using a combination of *in vivo* murine models, *in vitro* BMDC assays, and adoptive transfer approaches, we provide mechanistic evidence that DC-intrinsic NLRP3 signaling is indispensable for pollutant-driven exacerbation of allergic airway inflammation. These findings highlight a critical pathway through which environmental pollutants worsen asthma and identify NLRP3 as a potential therapeutic target for pollution-associated disease endotypes.

## Materials and methods

### Animals

Female C57BL/6J mice (6–8 weeks old) and Nlrp3 knockout (Nlrp3^−^/^−^) mice on a C57BL/6J background were obtained from The Jackson Laboratory (Bar Harbor, ME, USA). Mice were bred in-house under specific pathogen–free (SPF) conditions. All animals were housed under a 12-hour light/dark cycle with free access to food and water. All experiments were approved by the Institutional Animal Care and Use Committee at Shenzhen University (approval number: 2023A0035) and conducted in accordance with ARRIVE guidelines.

### Allergen and benzo[a]pyrene exposure protocol

Following established procedures (14), mice were sensitized and challenged using a modified airway inflammation protocol (**Supplementary Figure S1** in supplemental materials). House dust mite (HDM) extract (*Dermatophagoides pteronyssinus*; Greer Laboratories, Lenoir, NC) was administered intranasally (25 µg protein in 50 µL PBS) on days 0, 7, and 14. Benzo[a]pyrene (BaP; Sigma-Aldrich, St. Louis, MO) was co-administered intranasally at 10 µg/mouse in 50 µL corn oil/PBS emulsion, either alone or together with HDM, according to experimental groups. Control mice received vehicle (PBS or corn oil) only. Mice were analyzed 48 h after the final challenge.

### Airway hyperresponsiveness

Airway mechanics were assessed 24 h after the final challenge using a FlexiVent system (SCIREQ, Montreal, Canada). Mice were anesthetized with ketamine/xylazine, tracheostomized, and mechanically ventilated. Airway resistance was measured in response to escalating doses of aerosolized methacholine (0–50 mg/mL). Data were expressed as airway resistance (Rrs).

### Bronchoalveolar lavage and differential cell counts

BAL fluid was collected by instilling 1 mL PBS through the tracheal cannula. Total leukocytes were enumerated using a hemocytometer. Differential cell counts (macrophages, eosinophils, neutrophils, lymphocytes) were determined by cytospin and Diff-Quik staining (Siemens). At least 200 cells per slide were counted in a blinded manner.

### Histology

Lungs were inflation-fixed with 4% paraformaldehyde, paraffin-embedded, and sectioned (5 µm). Hematoxylin–eosin (H&E) staining was used to evaluate peribronchial and perivascular inflammation. Goblet cell hyperplasia and mucus production were assessed by Periodic Acid–Schiff (PAS) staining. Inflammation scores were assigned by blinded observers using a semiquantitative scale as following description:

#### Blinded scoring by a pathologist

Scoring is performed by a pathologist blinded to experimental groups to avoid bias. Evaluate 5–10 non-overlapping high-power fields (HPF; 400× magnification) containing bronchioles and associated blood vessels. Score the following key features:

#### Goblet cell hyperplasia

Using PAS-stained sections, quantify the proportion of goblet cells lining the bronchial epithelium. Score as:

0: No goblet cells.

1: <25% of epithelial cells are goblet cells.

2: 25–50% of epithelial cells are goblet cells.

3: 51–75% of epithelial cells are goblet cells.

4: >75% of epithelial cells are goblet cells.

### Serum IgE measurement

Blood was collected by cardiac puncture at sacrifice. Serum HDM-specific IgE was measured by ELISA (BD Biosciences) according to manufacturer’s instructions.

### Cytokine and alarmin measurement

Cytokines (IL-4, IL-5, IL-13, IL-17A, IL-1β, IL-18, TSLP, IL-33) were quantified in lung homogenates or BAL fluid using commercial ELISA kits (R&D Systems, Minneapolis, MN). Data were normalized to total protein concentration (BCA assay, Thermo Fisher Scientific).

### Bone marrow–derived dendritic cell culture and stimulation

BMDCs were generated from femurs and tibias of WT or Nlrp3^−^/^−^ mice. Cells were cultured in RPMI-1640 supplemented with 10% FBS, 2 mM L-glutamine, 50 µM β-mercaptoethanol, 1% penicillin/streptomycin, and 20 ng/mL GM-CSF (PeproTech). On day 7, BMDCs were stimulated with HDM (20 µg/mL), BaP (5 µM), or both for 24 h. Supernatants were collected for cytokine assays.

On Day 7, non-adherent and loosely adherent cells were gently harvested by pipetting, while firmly adherent cells (predominantly macrophages) were retained in the culture dish. This selection step enriches for BMDCs, as mature DCs in GM-CSF cultures typically exhibit non-adherent or loosely adherent properties, whereas bone marrow derived macrophages remain strongly adherent (15). To further confirm DC purity, flow cytometric analysis was performed to assess the expression of DC-specific markers (CD11c^+^) and macrophage markers (F4/80^+^), with BMDC preparations consistently showing >90% CD11c^+^ cells and <5% F4/80^+^ cells (representative gating strategy in **Supplementary Figure S1**).

### Flow cytometry

BMDCs were stained with fluorochrome-conjugated antibodies against CD80, CD86, and MHC-II (BioLegend). For T cell polarization assays, naïve CD4^+^ OT-II T cells were co-cultured with OVA-pulsed BMDCs for 5 days. CFSE dilution (for proliferation) and intracellular cytokine staining (IL-4, IL-17A, IFN-γ) were performed after PMA/ionomycin stimulation in the presence of brefeldin A. Data were acquired on a BD FACSCanto II and analyzed using FlowJo software. Gating strategy is illustrated in **Supplementary Figure S2**. Antibodies used in flow cytometry include IL-4 (Thermo Fisher Scientific, 17-7041-82, PE-Cyanine7), IL-5 (Thermo Fisher Scientific, 48-7052-82, eFluor™ 450), IL-13 (Thermo Fisher Scientific, 53-7133-82, Alexa Fluor^®^ 488), IL-17A (BD Biosciences, 561083, PE), TSLP (R&D Systems, FAB421P, Alexa Fluor^®^ 647), IL-33 (BioLegend, 510904, PE), IL-1β (BD Biosciences, 559367, FITC), IL-18 (Thermo Fisher Scientific, 12-7181-82, PerCP-eFluor™ 710), Caspase-1 (active) (ImmunoChemistry Technologies, 9501, FAM-FLICA), CD80 (BioLegend, 104712, PE-Cyanine5), CD86 (Elabscience, E-AB-F1012L, Elab Fluor^®^ 488).

### Adoptive transfer of lung DCs

DCs were isolated from lungs of WT or Nlrp3^−^/^−^ mice exposed to HDM+BaP using CD11c MicroBeads (Miltenyi Biotec). Purity (>90%) was confirmed by flow cytometry. A total of 1 × 10^6^ DCs were transferred intratracheally into naïve C57BL/6J recipient mice, which were subsequently challenged with HDM (25 µg intranasally on days 1 and 3). Recipients were analyzed on day 5 for AHR, BAL inflammation, and lung cytokines.

### BMDC-OVA-pulse assay

BMDCs were generated from the bone marrow of wild-type (WT) or Nlrp3-deficient (Nlrp3^−^/^−^) mice. On day 7 (at BMDC maturation stage), cells were harvested and adjusted to a density of 1×10^6^ cells/mL in complete RPMI medium. For OVA pulsing, BMDCs were incubated with 50 μM (16) endotoxin-free ovalbumin (OVA, Grade V; Sigma-Aldrich, Cat. No. A5503) at 37 °C with 5% CO_2_ for 4 hours. After pulsing, BMDCs were washed twice with phosphate-buffered saline (PBS) to remove unbound OVA, then pre-treated with Benzo[a]pyrene (BaP) for 24 hours. Subsequently, OVA-pulsed, BaP-pretreated BMDCs were co-cultured with naïve OT-II CD4^+^ T cells (isolated from OT-II transgenic mice via magnetic bead sorting, Miltenyi Biotec, Cat. No. 130-104-454) at a DC:T cell ratio of 1:10 for 72 hours, followed by analysis of T cell proliferation and cytokine production.

### Statistical analysis

Group comparisons were performed using one-way or two-way ANOVA with Tukey’s *post hoc* test or Student’s t-test, as appropriate. Nonparametric tests were used where data violated normality assumptions. p < 0.05 was considered statistically significant. Statistical analyses were conducted in R v4.5.1.

## Results

### Co-exposure to BaP exacerbates HDM-induced allergic airway inflammation

**Figure 1:**
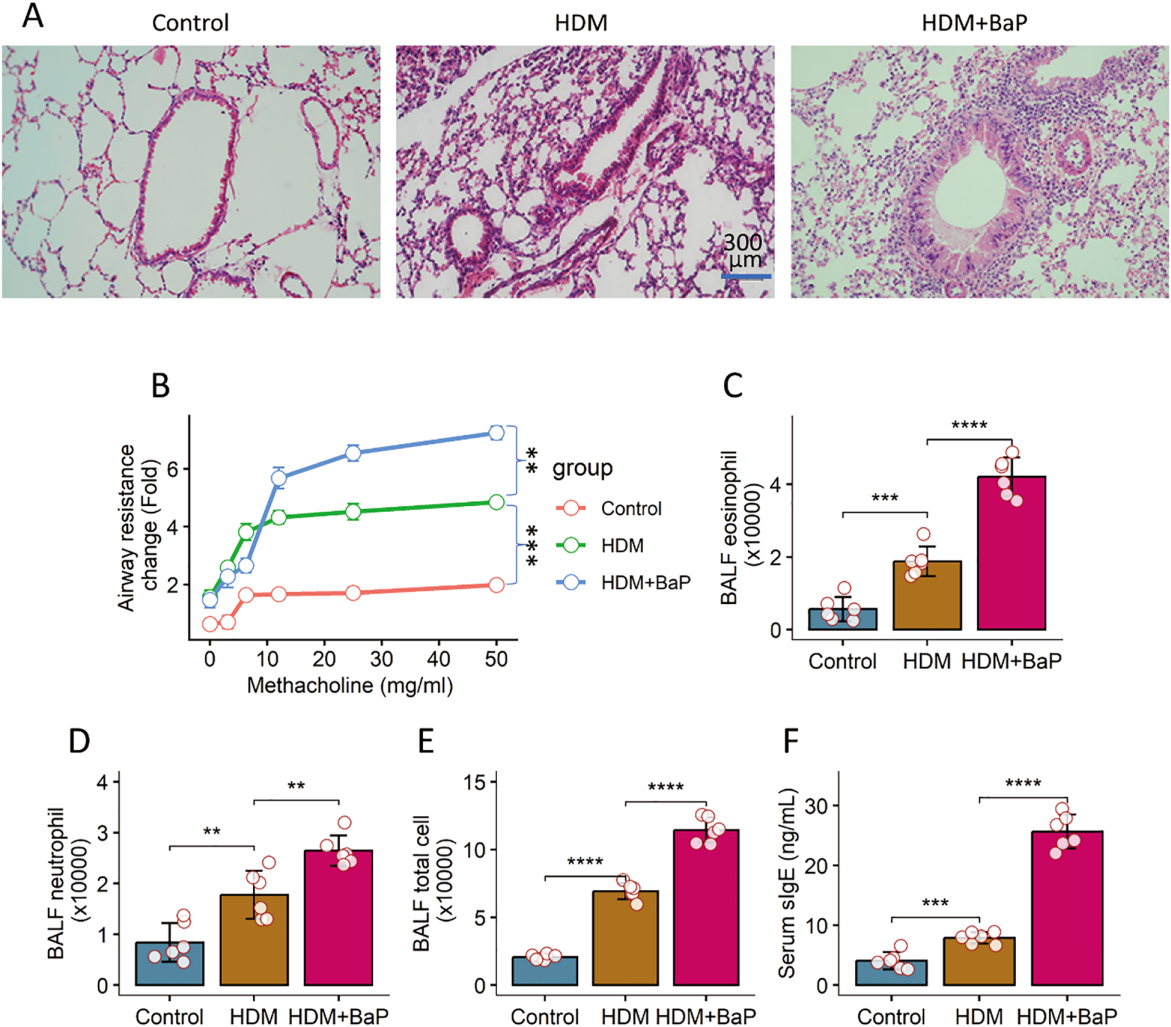
BaP co-exposure exacerbates HDM-induced allergic airway inflammation. **(A)** Representative histology images of lungs (×200). **(B)** Airway hyperresponsiveness (AHR) responses to methacholine challenge. **(C)** Bronchoalveolar lavage fluid (BALF) eosinophil counts. **(D)** BALF leukocyte counts. **(E)** BALF total leukocyte counts. **(F)** Serum levels of HDM-specific IgE. Each group consists of 6 mice. Each dot in bars presents one sample. Data of bar graph are presented as mean ± SD. Statistical significance for comparisons between groups was determined by one-way ANOVA followed by Tukey *post hoc* test. **p<0.01, ***p<0.001, ****p<0.0001.

To investigate the impact of pollutant exposure on allergen-induced asthma, we employed a sensitization and challenge protocol where mice were exposed to house dust mite (HDM) with or without benzo[a]pyrene (BaP) (**Supplementary Figure S1**). Co-exposure to HDM + BaP induced lung inflammation, manifesting dense peribronchial and perivascular inflammatory infiltration, goblet cell hyperplasia, and mucus hypersecretion (**Figure 1A**) and markedly increased airway hyperresponsiveness (AHR) in response to methacholine (*p* < 0.01; **Figure 1B**), consistent with epidemiological links between polycyclic aromatic hydrocarbons and worsened asthma severity (17). Bronchoalveolar lavage fluid (BALF) analysis revealed significantly elevated total leukocytes and a notable increase in eosinophils and neutrophils (*p* < 0.05, *p* < 0.001; **Figures 1C–E**). Furthermore, serum HDM-specific IgE levels were significantly greater in co-exposed mice than in HDM-only counterparts (*p* < 0.001; **Figure 1F**), reflecting pollutant-enhanced allergen sensitization (18).

### BaP co-exposure amplifies Th2/Th17 cytokine responses and epithelial alarmin expression

**Figure 2:**
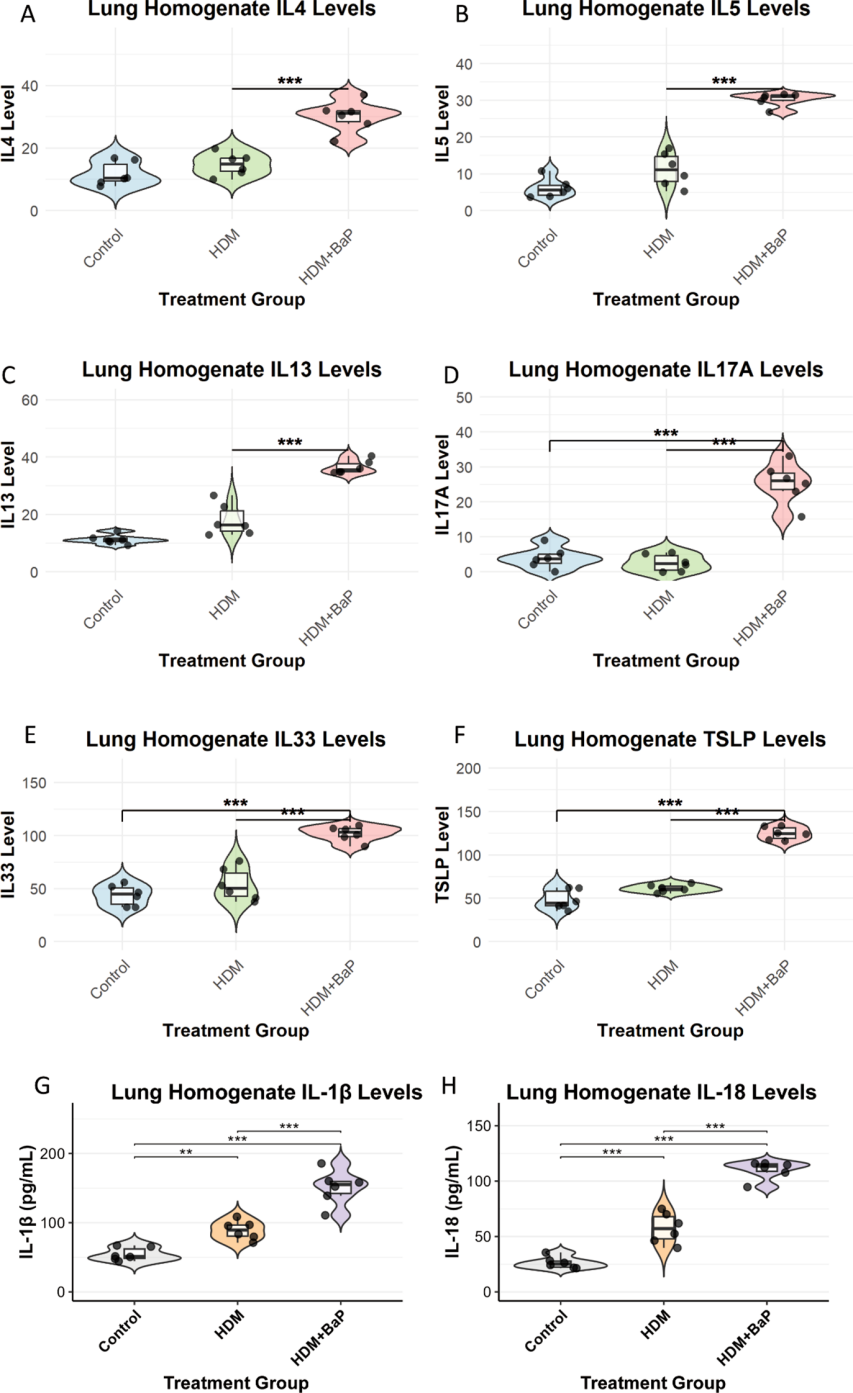
BaP co-exposure amplifies Th2/Th17 cytokine responses and epithelial alarmin expression in lung homogenates. Quantification of **(A)** IL-4, **(B)** IL-5, **(C)** IL-13 (Th2 cytokines), **(D)** IL-17A (Th17 cytokine), and **(E)** IL-33, **(F)** TSLP (epithelial alarmins), **(G, H)** IL-1β and IL-18 (proinflammatory cytokines) levels in lung homogenates from Control, House Dust Mite (HDM) challenged, and HDM + Benzo[a]pyrene (BaP) co-exposed mice. Each dot represents data from an individual mouse (n=6 mice per group). Data are presented as violin plots (displaying data distribution) overlaid with boxplots (showing median, 25th, and 75th percentiles) and jittered individual data points. Statistical significance was determined by one-way ANOVA followed by Tukey’s *post-hoc* test for multiple comparisons. Significant differences between groups are indicated by an asterisk: **p<0.01, ***p<0.001.

We next measured cytokine levels in lung homogenates. Th2 cytokines—IL-4, IL-5, and IL-13—were significantly elevated in the HDM + BaP group compared to HDM alone (*p* < 0.05, *p* < 0.01; **Figures 2A–C**). Notably, IL-17A (Th17 cytokine) was strongly induced exclusively in the co-exposure group (**Figure 2D**), supporting a pollutant-induced shift toward mixed Th2/Th17 inflammation (19). Epithelial alarmins—TSLP and IL-33—were also significantly upregulated in co-exposed lungs (*p* < 0.001; **Figures 2E, F**), consistent with alarmin-mediated amplification of type 2 responses (20).

### The exacerbated allergic phenotype is dependent on the NLRP3 inflammasome

**Figure 3:**
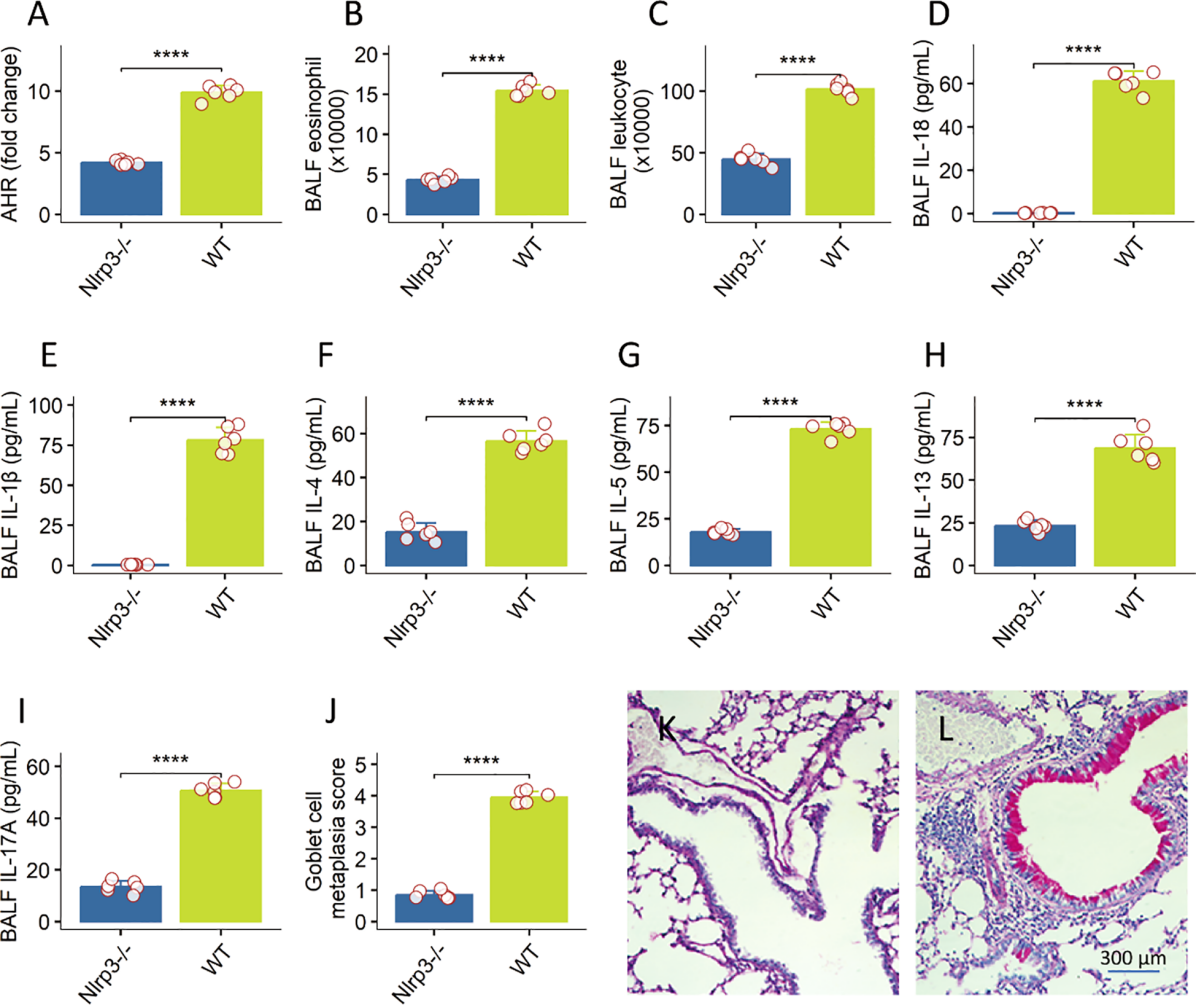
NLRP3 inflammasome is essential for pollutant-driven asthma exacerbation in a murine model. data representing various parameters of allergic airway inflammation were generated for Wild-type (WT) and Nlrp3-deficient (Nlrp3^−/−^) mice subjected to a HDM + BaP protocol, with 6 mice per group. **(A)** Airway Hyperresponsiveness (AHR): Nlrp3 deficiency significantly mitigated AHR, reflecting reduced airway constriction in response to methacholine challenge (the value was recorded at the last dose: 50 mg/mL). **(B, C)** BALF Total Leukocytes and Eosinophils: Nlrp3^−/−^ mice showed significantly reduced total leukocyte counts and notably diminished eosinophil numbers in bronchoalveolar lavage fluid (BALF). **(D–I)** BALF Cytokines: Signature NLRP3-activated cytokines, IL-1β and IL-18, as well as the Th2 cytokines, were virtually undetectable in the BALF of Nlrp3^−/−^ mice, in contrast to WT controls. **(J–L)** Goblet Cell Metaplasia: Quantification of goblet cells in lung sections demonstrated a substantial diminution of goblet cell metaplasia in the absence of Nlrp3 **(J)**. **(K, L)**, representative histology images show goblet cell metaplasia in the lung. Data are presented as mean ± Standard Error of the Mean (SEM). Individual mouse data points are shown as jittered dots. Statistical comparisons between WT and Nlrp3^−/−^ groups for each parameter were performed using unpaired Welch’s *t*-tests. *P*-values are indicated as: ****p < 0.0001. These findings highlight the critical role of the NLRP3 inflammasome in exacerbating allergic airway inflammation.

To probe the role of innate immune sensing, we compared wild-type (WT) and Nlrp3^−^/^−^ mice under the HDM + BaP protocol. Nlrp3 deficiency significantly mitigated AHR (*p* < 0.01; **Figure 3A**) and reduced BALF leukocytes, particularly eosinophils (**Figure 3B**). IL-1β and IL-18—signature NLRP3-activated cytokines—were virtually undetectable in Nlrp3^−^/^−^ BALF (**Figures 3C–I**). Goblet cell metaplasia was also substantially diminished in Nlrp3-deficient lungs (**Figures 3J–L**). These data highlight NLRP3 as essential for pollutant-driven asthma exacerbation, echoing findings from NLRP3’s role in allergic models (21).

### BaP potentiates NLRP3 inflammasome activation and functional maturation in dendritic cells

**Figure 4:**
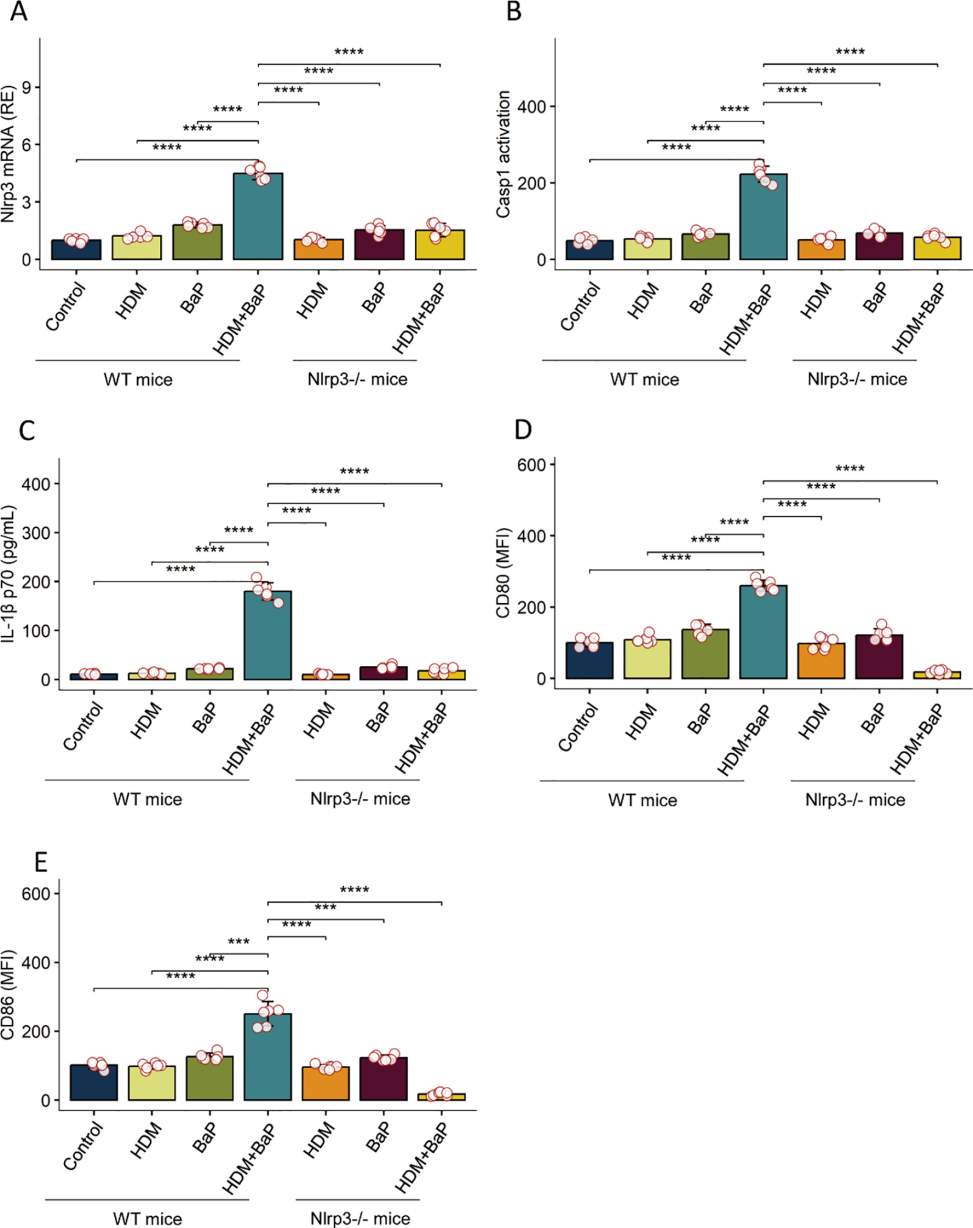
Benzo[a]pyrene (BaP) potentiates HDM-induced NLRP3 inflammasome activation and dendritic cell maturation in BMDCs.Bone marrow-derived dendritic cells (BMDCs) from wild-type (WT) and Nlrp3-deficient (Nlrp3^-/-^) mice were stimulated as indicated: Control (Cntrl), house dust mite (HDM), Benzo[a]pyrene (BaP), or a combination of HDM+BaP. The panels display: **(A)** NLRP3 mRNA expression (relative to control), **(B)** Caspase-1 activation (FLICA arbitrary units, AU), **(C)** mature IL-1β p17 secretion (pg/mL), **(D)** surface CD80 expression (Mean Fluorescence Intensity, MFI), and **(E)** surface CD86 expression (MFI). In WT BMDCs, combined HDM+BaP stimulation substantially increased NLRP3 expression, Caspase-1 activation, IL-1β p17 secretion, and upregulation of co-stimulatory molecules (CD80 and CD86) compared to untreated controls or single treatments. Crucially, this potentiated response was abrogated in Nlrp3^-/-^ BMDCs, demonstrating an essential role for NLRP3 in the observed effects. Data are presented as mean + standard error of the mean (SEM) with individual data points (n=6 mice per group). Statistical significance was assessed using one-way ANOVA followed by Tukey’s *post hoc* test for multiple comparisons; ***p < 0.001, ****p< 0.0001. Representative flow cytometry plots for panels D and E are presented in **Supplementary Figure S4**.

We next assessed whether dendritic cells (DCs) are direct targets of BaP. In BMDCs, co-treatment with HDM + BaP significantly enhanced expression of NLRP3, caspase-1 activation, and secretion of mature IL-1β p17 (**Figures 4A–C**, **Supplementary Figure S3**). Co-exposure also elevated surface expression of CD80 and CD86 (**Figures 4D, E**, **Supplementary Figure S4**), indicating DC maturation. These responses were absent in Nlrp3^−/−^ BMDCs, confirming that BaP potentiates NLRP3-dependent DC activation.

### NLRP3-dependent dendritic cells are sufficient to drive allergic airway inflammation

**Figure 5:**
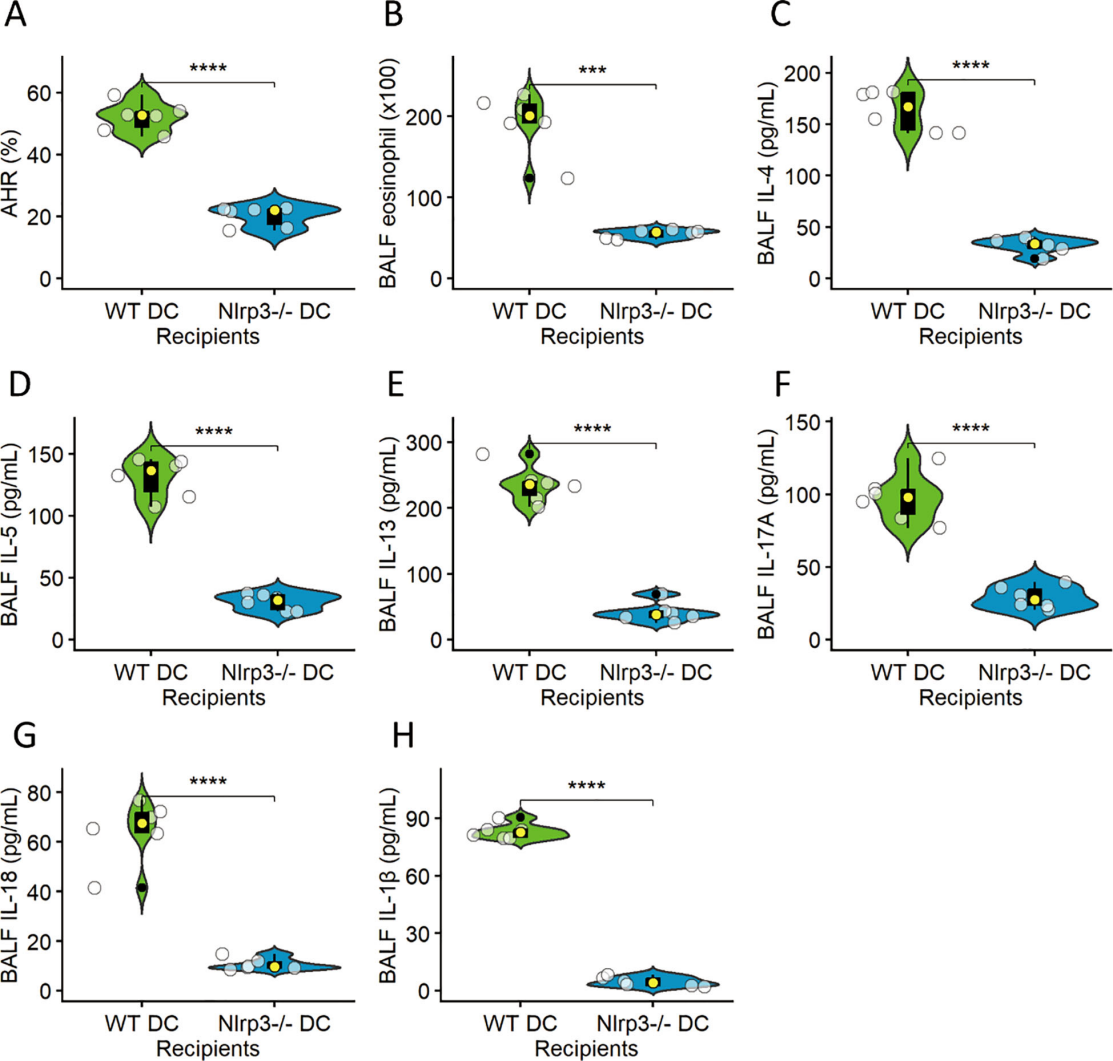
NLRP3-dependent dendritic cells are sufficient to drive allergic airway inflammation. Lung dendritic cells (DCs) isolated from HDM+BaP-treated wild-type (WT) or Nlrp3-deficient (Nlrp3^−^/^−^) mice were adoptively transferred into naïve recipients, followed by an HDM challenge. The panels illustrate various hallmarks of allergic airway inflammation in these recipient mice: **(A)** Airway hyperresponsiveness (AHR), **(B)** Eosinophilic inflammation (quantified as eosinophils per BALF), **(C)** IL-4, **(D)** IL-5, **(E)** IL-13, **(F)** IL-17A, **(G)** IL-18 and **(H)** IL-1β levels (all Th2/Th17 cytokines measured in lung homogenates in pg/mL). Recipients of WT DCs developed robust AHR, significant eosinophilic inflammation, and elevated Th2 (IL-4, IL-5, IL-13) and Th17 (IL-17A) cytokine levels in the lung. In stark contrast, Nlrp3^−^/^−^ DC recipients exhibited significantly attenuated responses across all measured parameters, indicating that NLRP3 expression within DCs alone is critical and sufficient to orchestrate allergen-driven immunopathology. Data are presented as median (IQR) with individual data points (n=6 mice per group). Statistical significance was assessed using independent t-tests; ***p < 0.001, ****p< 0.0001.

To test sufficiency, lung DCs from HDM + BaP–treated WT or Nlrp3^−^/^−^ mice were adoptively transferred into naïve recipients, followed by HDM challenge. Recipients of WT DCs developed robust AHR (**Figure 5A**), eosinophilic inflammation (**Figure 5B**), and elevated Th2/Th17 cytokines in lung homogenates (**Figures 5C–F**). In contrast, Nlrp3^−^/^−^ DC recipients exhibited significantly attenuated responses (**Figure 5**), indicating that NLRP3 in DCs alone can orchestrate allergen-driven immunopathology (22).

### NLRP3 signaling in DCs drives pathogenic T helper cell polarization

**Figure 6:**
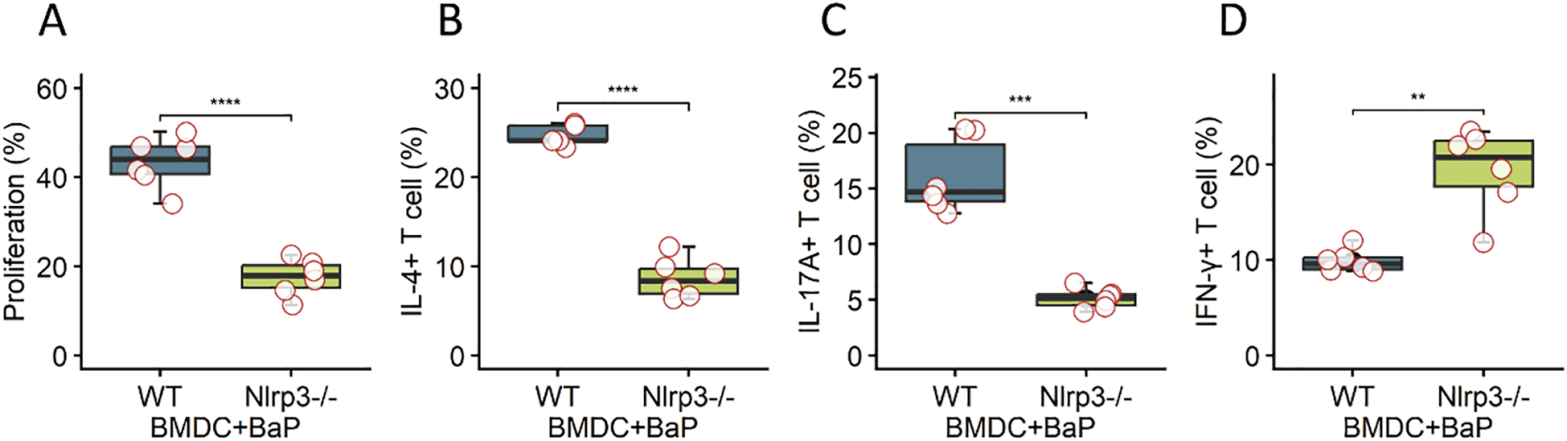
DC-intrinsic NLRP3 signaling drives pathogenic T helper cell polarization. Ovalbumin (OVA)-pulsed bone marrow-derived dendritic cells (BMDCs) obtained from wild-type (WT) or Nlrp3-deficient (Nlrp3^−^/^−^) mice were pre-treated with Benzo[a]pyrene (BaP) and then co-cultured with OT-II CD4^+^ T cells. The panels demonstrate key T cell responses: **(A)** Percentage of proliferating T cells, **(B)** Percentage of IL-4^+^ (Th2) T helper cells, **(C)** Percentage of IL-17A^+^ (Th17) T helper cells, and **(D)** Percentage of IFN-γ^+^ (Th1) T helper cells. Co-culture with WT BMDCs plus BaP significantly promoted T cell proliferation and skewed T cell differentiation toward pathogenic Th2 (IL-4^+^) and Th17 (IL-17A^+^) subsets, accompanied by a concurrent reduction in IFN-γ^+^ Th1 cells. Crucially, this pathogenic T helper cell phenotype and enhanced proliferation were not observed when OT-II CD4^+^ T cells were co-cultured with Nlrp3^−^/^−^ BMDCs. These findings collectively establish DC-intrinsic NLRP3 as a critical link connecting exposure to environmental pollutants (BaP) with the subsequent pathogenic T helper cell polarization. Data are presented as mean + standard error of the mean (SEM) with individual data points (n=6 mice per group). Statistical significance was assessed using independent *t*-tests; ****p<0.0001, ***p <0.001, **p< 0.01.

Finally, we examined T cell priming by NLRP3-activated DCs. OVA-pulsed BMDCs from WT or Nlrp3^−^/^−^ mice treated with BaP were co-cultured with OT-II CD4^+^ T cells. Co-culture with WT BMDCs plus BaP promoted T cell proliferation (**Figure 6A**) and skewed differentiation toward IL-4^+^ Th2 and IL-17A^+^ Th17 subsets, with a concurrent reduction in IFN-γ^+^ Th1 cells (**Figures 6B, C**). This phenotype was not observed with Nlrp3^−^/^−^ BMDCs (**Figure 6D**). These findings establish DC-intrinsic NLRP3 as a critical link between environmental pollutant exposure and pathogenic T helper polarization (23).

## Discussion

In this study, we demonstrate that environmental exposure to the polycyclic aromatic hydrocarbon (PAH) benzo[a]pyrene (BaP) markedly exacerbates HDM-induced allergic airway inflammation in mice. Co-exposure led to heightened airway hyperresponsiveness, intensified eosinophilic and neutrophilic infiltration, enhanced mucus hypersecretion, and elevated allergen-specific IgE. Mechanistically, BaP amplified both Th2 and Th17 cytokine responses, induced epithelial alarmins, and critically engaged the NLRP3 inflammasome pathway. Functional studies *in vivo* and *in vitro* established that NLRP3 activation in dendritic cells (DCs) is indispensable for the pollutant-driven exacerbation of allergic airway inflammation and the polarization of pathogenic Th2/Th17 responses. Together, these findings provide mechanistic insights into how environmental pollutants potentiate asthma severity through innate immune–adaptive immune crosstalk.

### Environmental pollutants as amplifiers of allergic airway disease

Epidemiological and experimental evidence has long suggested that air pollutants, particularly PAHs from traffic emissions and cigarette smoke, worsen asthma severity and prevalence (17). Our study extends these observations by providing direct mechanistic evidence that BaP synergizes with allergen exposure to drive exaggerated airway inflammation. Importantly, we found that BaP not only magnifies Th2-mediated eosinophilia but also introduces a robust Th17 component, resulting in mixed granulocytic inflammation. This mixed Th2/Th17 endotype is increasingly recognized as a hallmark of severe, steroid-resistant asthma (24). Thus, our data link environmental pollutant exposure to immune pathways implicated in difficult-to-treat asthma.

### NLRP3 inflammasome as a central hub

A key discovery is the requirement for the NLRP3 inflammasome in mediating BaP-driven pathology. Nlrp3-deficient mice exhibited marked protection against airway hyperresponsiveness, leukocyte infiltration, goblet cell metaplasia, and type 2 cytokine production. These findings are consistent with emerging reports implicating the NLRP3–IL-1 axis in allergic airway disease (25). By demonstrating that pollutant-driven allergen responses are abrogated in Nlrp3^−/−^ mice, our results highlight NLRP3 as a key convergence point between environmental toxicants and allergen-induced immune activation.

### Dendritic cells as pollutant sensors and immune amplifiers

We further identified DCs as critical cellular mediators of this pathway. BaP co-stimulation enhanced NLRP3 activation and IL-1β release in BMDCs, promoted their maturation, and potentiated their ability to prime pathogenic Th2 and Th17 responses. Adoptive transfer experiments confirmed that DCs from BaP/HDM-exposed lungs are sufficient to transfer exacerbated allergic inflammation in an NLRP3-dependent manner. These findings build on prior work demonstrating that DCs integrate environmental signals and orchestrate adaptive immunity (26). Our results specifically implicate DC-intrinsic NLRP3 as the link between pollutant exposure and maladaptive T cell polarization.

### Integration with epithelial alarmin signaling

We also observed that BaP exposure boosted production of epithelial alarmins (TSLP, IL-33), which are central to allergen-induced type 2 inflammation (27). It is possible that BaP exposure primes both structural cells (airway epithelium) and immune cells (DCs), creating a feed-forward circuit that amplifies allergic inflammation. Whether NLRP3 activation in epithelial cells also contributes remains to be clarified, but our data emphasize DCs as indispensable effectors.

### Implications for asthma endotypes and therapy

Our findings have significant clinical implications. First, they provide a mechanistic explanation for why individuals in high-pollution environments may develop more severe or treatment-resistant asthma phenotypes. Second, they identify the NLRP3 inflammasome as a potential therapeutic target for pollutant-exacerbated asthma. Pharmacological inhibition of NLRP3 or IL-1 signaling is currently under investigation for inflammatory diseases (28), and our data suggest these strategies could be extended to pollution-associated asthma endotypes.

### Diverse mechanisms of IL-33 release

IL-33, an alarmin, is predominantly stored in and released from stressed or damaged structural cells, notably airway epithelial cells, during inflammation. Its release can occur via several pathways, many of which are caspase-1-independent. These include necrotic cell death, necroptotic cell death, or simple cellular stress that leads to IL-33 secretion without requiring specific proteolytic cleavage. In our model, HDM antigen itself can induce epithelial stress and damage, and the co-exposure to Benzo[a]pyrene (BaP), an environmental pollutant known to cause oxidative stress and cellular dysfunction, would significantly exacerbate epithelial injury and favor these caspase-1-independent release mechanisms, leading to a net increase in extracellular IL-33.

### Context-dependent caspase-1 cleavage of IL-33

While caspase-1 can indeed cleave IL-33, the functional consequence is complex and context-dependent. Some studies suggest caspase-1 cleavage results in inactivation, while others demonstrate that caspase-1 can generate shorter, processed forms of IL-33 that retain or even exhibit enhanced pro-inflammatory biological activity compared to the full-length molecule (e.g., [cite a relevant review or study, e.g., on distinct IL-33 isoforms and their bioactivities, like reviews by Liew et al. or studies by Lüthi et al. if applicable]). Therefore, an increase in total or bioactive extracellular IL-33 is not necessarily contradictory to inflammasome activation. It is plausible that the unique molecular environment orchestrated by HDM and BaP in the airways leads to forms of IL-33 processing that maintain its alarm-like function.

### Temporal and spatial dynamics

It is also important to consider the dynamics of inflammation. The release of IL-33 from compromised cells might occur concurrently with or even precede the full activation of inflammasomes in specific immune cell populations, or from different cellular sources. The cumulative effect of increased epithelial stress due to HDM and the additional damaging effects of BaP likely overwhelms any potential caspase-1 mediated inactivation of a subset of IL-33, leading to the observed overall elevation.

In fact, rather than a contradiction, the co-elevation of IL-33 (an alarmin) alongside inflammasome-derived IL-1β and IL-18 (both innate inflammatory cytokines) provides a more comprehensive picture of the robust innate immune activation and cellular distress occurring in our HDM+BaP murine model. This suggests multiple innate inflammatory pathways are simultaneously engaged to drive the Th2-pattern inflammatory response.

### Consideration of using female mice in the experiments

Female C57BL/6 mice (6–8 weeks old) were used for all allergic asthma experiments. This choice was guided by experimental evidence. Published studies consistently report that male mice exhibit higher baseline variability in allergic asthma phenotypes (e.g., airway hyperresponsiveness [AHR], eosinophilic inflammation, and Th2 cytokine production) compared to females in house dust mite (HDM)- or ovalbumin (OVA)-induced asthma models (29). Female mice display more reproducible and robust allergic responses to common asthma triggers, which minimized inter-animal variation.

### Limitations and future directions

Several limitations should be noted. We focused on BaP as a prototypical PAH, but real-world exposures involve complex pollutant mixtures, including diesel exhaust particles, ozone, and fine particulates. Future studies should examine whether similar NLRP3-dependent mechanisms apply across diverse pollutants. Additionally, while our findings in mice and BMDCs provide mechanistic insights, validation in human cells and clinical samples will be essential. Finally, the interplay between NLRP3 and other inflammasome pathways (e.g., AIM2, NLRC4) warrants exploration.

## Conclusion

In summary, we show that BaP, a representative environmental pollutant, exacerbates allergen-induced asthma through NLRP3-dependent activation of dendritic cells, leading to Th2/Th17 polarization and severe airway inflammation. These findings mechanistically link pollutant exposure to pathogenic asthma endotypes and suggest that targeting NLRP3 may offer novel therapeutic opportunities for patients with environmentally aggravated asthma.

## Data Availability

The original contributions presented in the study are included in the article/**Supplementary Material**. Further inquiries can be directed to the corresponding authors.
