# Supplementary material for: Benzo[a]pyrene exacerbates allergen-induced airway inflammation through NLRP3-dependent dendritic cell activation and pathogenic T helper cell polarization
Source: Front Immunol. 2025 Nov 19;16:1699886. doi: 10.3389/fimmu.2025.1699886 (PMC12672459; doi:10.3389/fimmu.2025.1699886)
Supplement: Supplementary file 1 [file DataSheet1.docx]

**Supplemental materials**


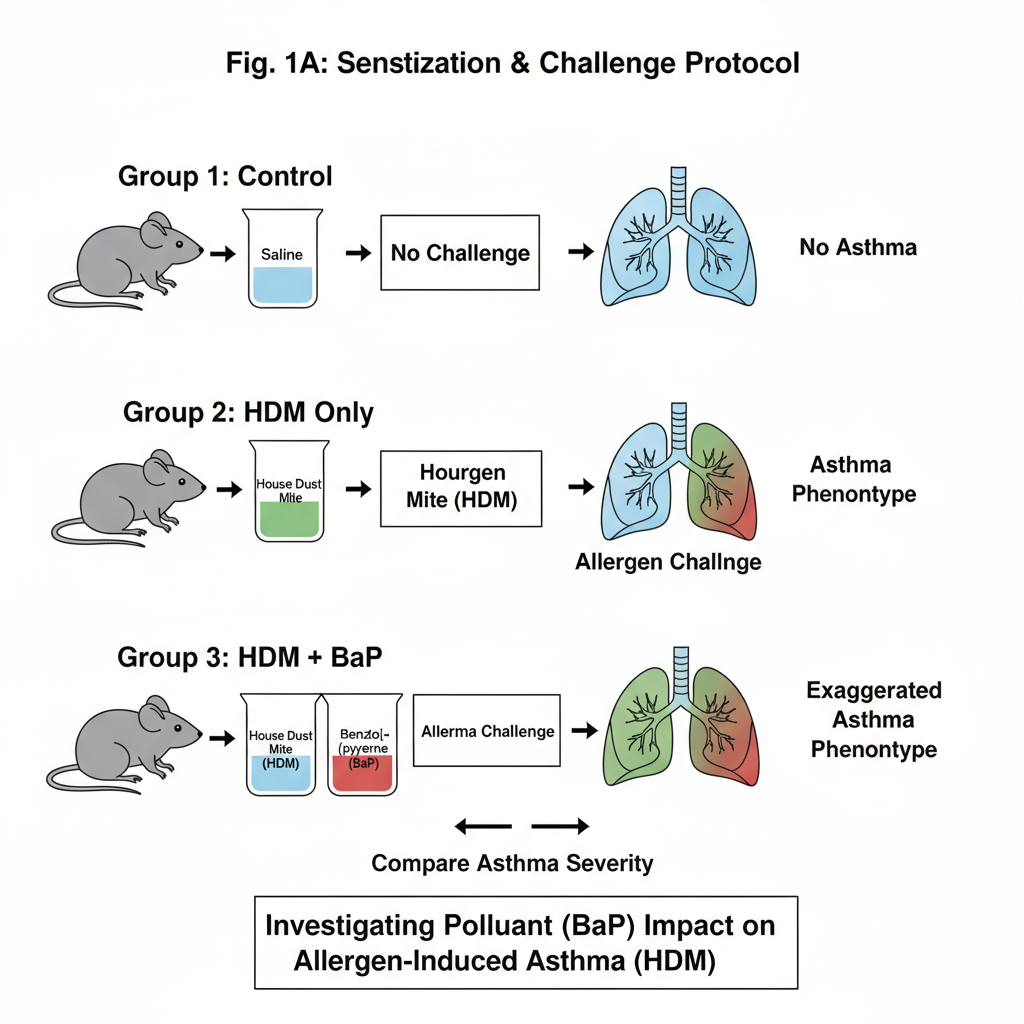


**Figure S1. Outlines a sensitization and challenge protocol designed to investigate the impact of Benzol(a)pyrene (BaP) on allergen-induced asthma**.

- **Group 1: Control** mice receive saline, undergo no challenge, and consequently show no asthma.
- **Group 2: HDM Only** mice are sensitized with House Dust Mite (HDM) and then subjected to an allergen challenge, resulting in an asthma phenotype.
- **Group 3: HDM + BaP** mice are sensitized with both House Dust Mite (HDM) and Benzol(a)pyrene (BaP), followed by an allergen challenge, leading to an exaggerated asthma phenotype.

The protocol aims to compare asthma severity across the groups to understand how pollutants like BaP influence allergen-induced asthma.


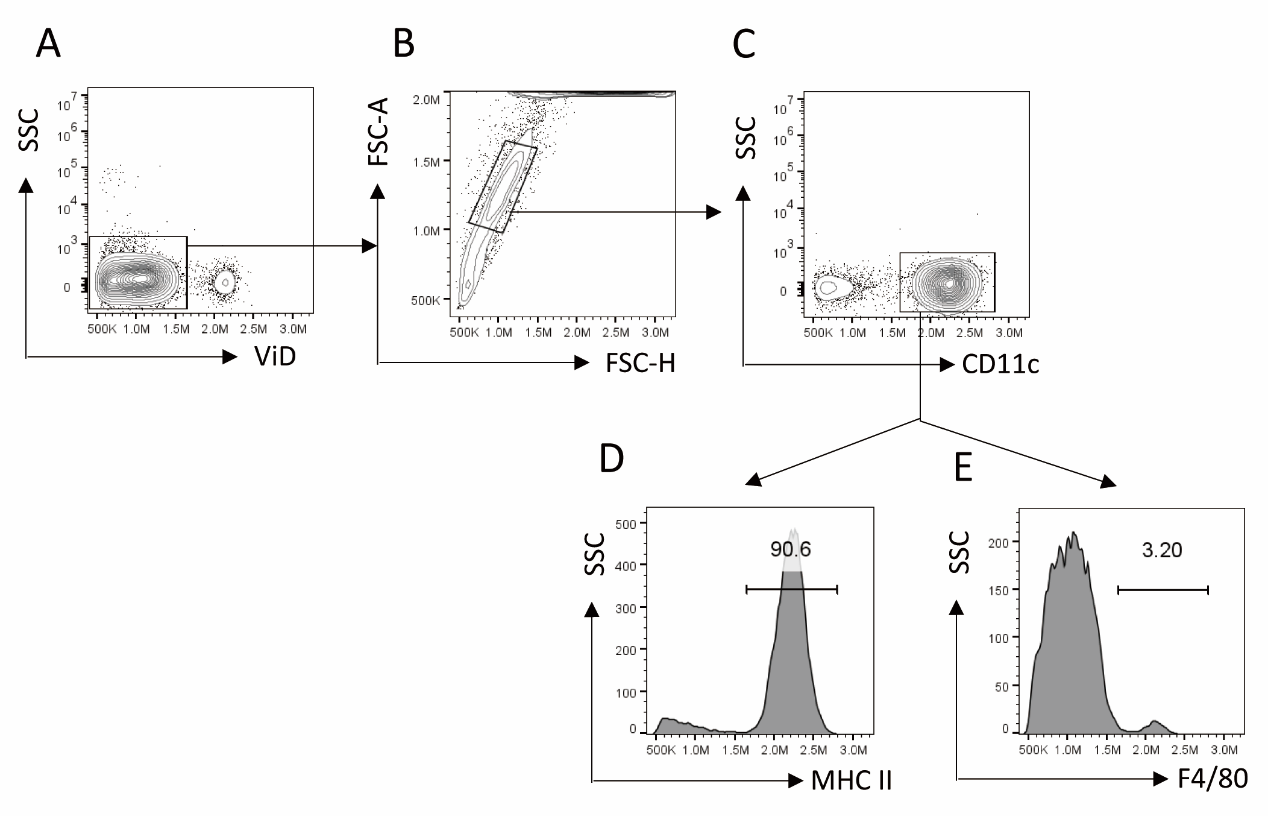


**Figure S2. Gating Strategy for Bone Marrow-Derived Dendritic Cells (BMDCs)**

(A–B) Dead cells and outliers were excluded from the analysis. (C) From the remaining viable cell population, CD11c⁺ cells were gated. Subsequent gating within the CD11c⁺ population identified (D) MHC II⁺ cells (dendritic cells) and (E) F4/80⁺ cells (macrophages). ViD: Viable cell staining dye.


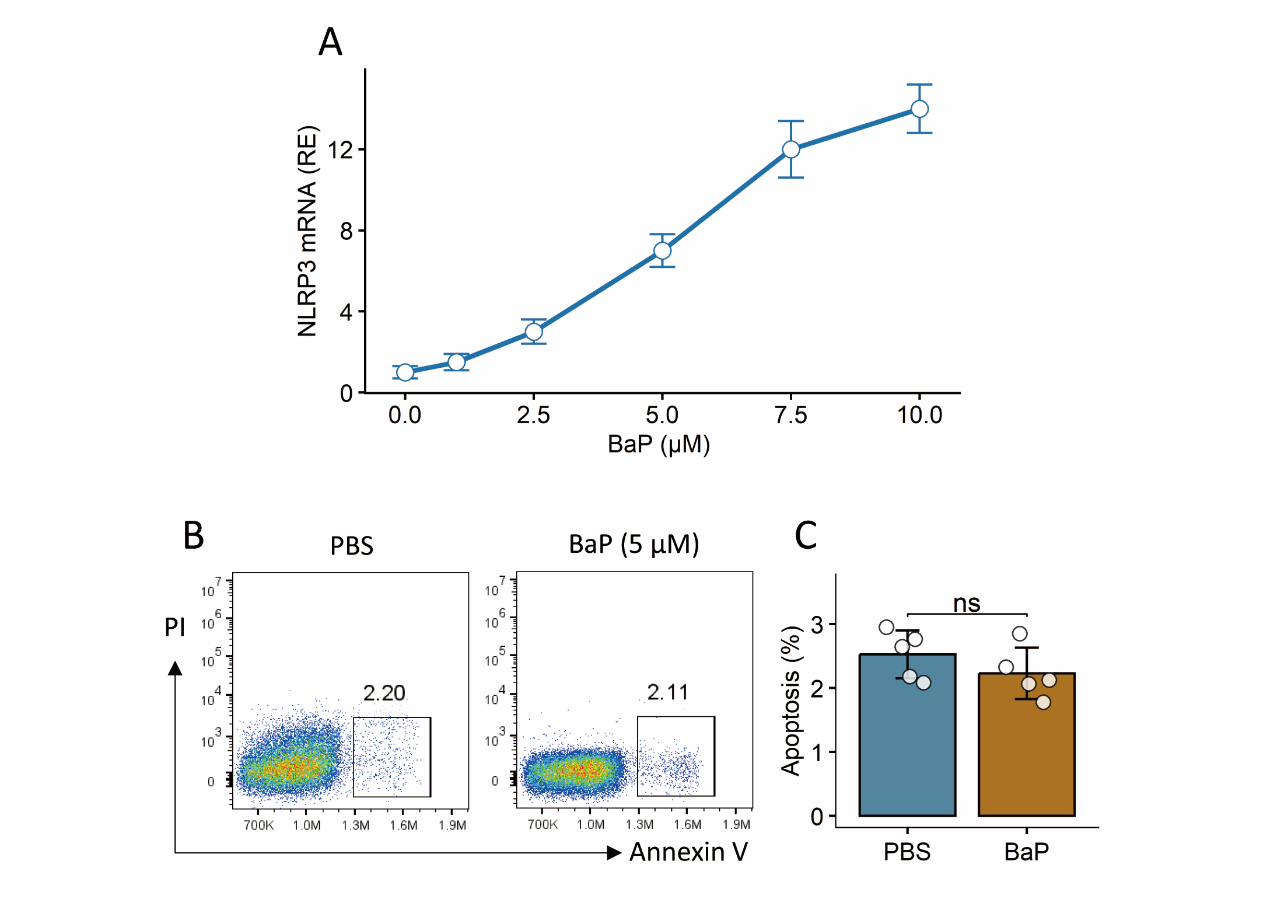


**Figure S3. Optimization of Benzo[a]pyrene (BaP) Dosage for Cell Culture Experiments**

(A) NLRP3 mRNA expression in bone marrow-derived dendritic cells (BMDCs) following 24-hour exposure to BaP at the indicated concentrations.

(B) Representative flow cytometry plots showing apoptotic BMDCs after BaP treatment (5 µM).

(C) Bar graphs depict the mean ± standard deviation (SD) of apoptotic BMDC frequencies from five independent experiments. Statistical significance was determined by Student’s t-test; ns = not significant.


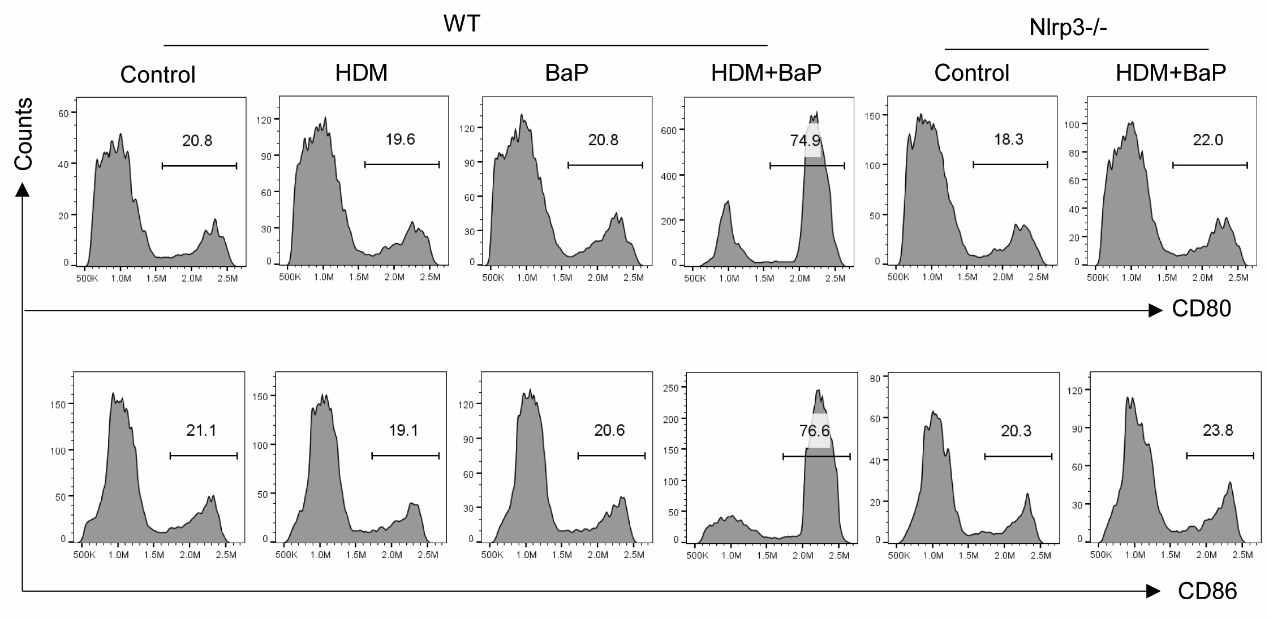


**Figure S4. Assessment of CD80 and CD86 Expression in Bone Marrow-Derived Dendritic Cells (BMDCs)**

Bone marrow-derived dendritic cells (BMDCs) were isolated, prepared, and exposed to house dust mite (HDM), benzo[a]pyrene (BaP), or their combination in culture for 24 hours. Subsequent analysis of BMDCs was performed using flow cytometry. Gated histograms depict the frequency of CD80-positive (CD80⁺) or CD86-positive (CD86⁺) BMDCs. Quantitative data corresponding to the percentage of CD80⁺ or CD86⁺ BMDCs are presented in Figure 4D and 4E.
